# Improved Alveolar Dynamics and Structure After Alveolar Epithelial Type II Cell Transplantation in Bleomycin Induced Lung Fibrosis

**DOI:** 10.3389/fmed.2021.640020

**Published:** 2021-02-17

**Authors:** Elena Lopez-Rodriguez, Gemma Gay-Jordi, Lars Knudsen, Matthias Ochs, Anna Serrano-Mollar

**Affiliations:** ^1^Institute of Functional Anatomy, Charité - Universitaetsmedizin Berlin, Berlin, Germany; ^2^Institute of Functional and Applied Anatomy, Hannover Medical School, Hannover, Germany; ^3^Experimental Pathology Department, Institut d'Investigacions Biomèdiques de Barcelona, Consejo Superior de Investigaciones Cientificas (IIBB-CSIC) Barcelona, Institut d'Investigacions Biomédiques August Pi i Sunyer (IDIBAPS), Barcelona, Spain; ^4^Centro de Investigaciones Biomédicas en Red de Enfermedades Respiratorias (CIBERES), Madrid, Spain; ^5^Biomedical Research in Endstage and Obstructive Lung Disease Hannover (BREATH), Member of the German Center for Lung Research (DZL), Hannover, Germany; ^6^German Center for Lung Research (DZL), Berlin, Germany

**Keywords:** lung fibrosis, alveolar epithelial type 2 cells, lung surfactant, cell therapy, bleomycin, alveolar dynamics, lung structure

## Abstract

Idiopathic pulmonary fibrosis (IPF) is a progressively and ultimately fatal lung disease. Previously it has been shown that intratracheal administration of alveolar epithelial type II cells (AE2C) in the animal model of bleomycin-induced pulmonary fibrosis is able to reverse fibrosis and restore surfactant protein levels. However, to date, it has not been evaluated whether these changes involve any improvement in alveolar dynamics. Consequently, the aim of the present work was to study lung physiology after AE2C transplantation at different time points during the development of injury and fibrosis. Lung fibrosis was induced by intratracheal instillation of bleomycin (4U/kg) in rat lungs. The animals were transplanted with AE2C (2.5 × 10^6^ cells/animal) 3 or 7 days after bleomycin instillation. Assessments were done at day 7 and 14 after the induction of fibrosis to plot time dependent changes in lung physiology and mechanics. To assess the pressures and rates at which closed alveoli reopens invasive pulmonary tests using a small-animal mechanical ventilator (Flexivent®, Scireq, Canada) including de-recruitability tests and forced oscillation technique as well as quasi-static pressure volume loops were performed. Afterwards lungs were fixed by vascular perfusion and subjected to design-based stereological evaluation at light and electron microscopy level. AE2C delivered during the lung injury phase (3 days) of the disease are only able to slightly recover the volume of AE2C and volume fraction of LB in AE2C. However, it did not show either positive effects regarding ventilated alveolar surface nor any increase of lung compliance. On the other hand, when AE2C are delivered at the beginning of the fibrotic phase (7 days after bleomycin instillation), an increased ventilated alveolar surface to control levels and reduced septal wall thickness can be observed. Moreover, transplanted animals showed better lung performance, with increased inspiratory capacity and compliance. In addition, a detailed analysis of surfactant active forms [mainly tubular myelin, lamellar body (LB)-like structures and multilamellar vesicles (MLV)], showed an effective recovery during the pro-fibrotic phase due to the healthy AE2C transplantation. In conclusion, AE2C transplantation during fibrogenic phases of the disease improves lung performance, structure and surfactant ultrastructure in bleomycin-induced lung fibrosis.

## Introduction

Idiopathic pulmonary fibrosis (IPF) is a progressive and severe disease with no known cause and with a limited response to currently available therapies, ultimately IPF is a fatal lung disease (1–3). The median survival time is 3–5 years from the time of diagnosis (1). The classic features of the disorder include progressive dyspnea and a non-productive cough. Pulmonary function tests usually reveal decreased lung volumes (especially decreased forced vital capacity, total lung capacity, and functional residual capacity) and diminished carbon monoxide diffusing capacity. During the course of the disease patients show a progressive decline in pulmonary function leading to respiratory failure and death.

The pathogenesis of IPF is characterized primarily by epithelial cell damage and inadequate regeneration. In normal physiological conditions, the renewal of alveolar epithelial cells occurs through the specific proliferation and differentiation of alveolar epithelial type 2 cells (AE2C) into alveolar type 1 cells. However, IPF is characterized by the loss of both alveolar cell types leading over time to epithelial necrosis, the appearance of fibroblast foci and persistent alveolar collapse (4, 5). In addition to AE2C dysfunction (5–7), IPF is also characterized by impaired surfactant function (8). In this sense, it is important to note that AE2C are also the cells responsible for synthesizing, storing, secreting and recycling the components of surfactant (9, 10) and therefore also play a crucial role in pulmonary mechanics by stabilizing alveolar dimensions and surface throughout the respiratory cycle (11, 12). During fibrosis development the surfactant dysfunction and edema increase the degree of alveolar recruitment and de-recruitment (alveolar R/D). The localized mechanical stresses imparted on the alveolar epithelium during R/D aggravate lung injury (13) leading to fibrotic remodeling (14, 15). The surface tension in some collapsed alveoli may become so high that recruitment is impossible. Eventually, collapse induration can occur whereby chronically collapsed alveoli effectively disappear by being reabsorbed into the surrounding interstitial tissue increasing the damage (5, 16, 17).

Since AE2C seem to be key cells in the fibrotic development, it has been proposed that re-generation or replacement of AE2C may be an alternative for the therapy of lung fibrosis patients (18–20). In this context, transplanting healthy donor AE2C in fibrotic lungs is a promising tool to explore. Previously, our research group has shown that intratracheal administration of AE2C in the animal model of bleomycin-induced pulmonary fibrosis was able to reverse fibrosis and restores surfactant protein levels (18, 19). Although our research group pioneered the development of this cell therapy, our results have also been corroborated by other research groups. They have also observed that both AE2C and stem cells derived to AE2C have also been able to reverse pulmonary fibrosis (20–24). Moreover, in a clinical study performed with IPF patients, the intratracheal administration of heterologous AE2C was safe, well-tolerated, with no relevant side effects, and was able to stabilize disease progression, improving health-related quality of life throughout a 1-year clinical follow-up (25). Those astonishing results obtained in humans were assessed by means of non-invasive pulmonary function tests, however to date it has not been evaluated whether these changes are related to any improvement in lung dynamics and structure. Consequently, the aim of the present work was to study lung physiology after AE2C transplantation at different time points during the development of fibrosis.

## Methods

### Animals

Fischer 344 rats, weighting 200–225 g at the beginning of the experiment, were used, in accordance with the European Community (Directive 2010/63/EU) for experimental animals and it was approved by the local authorities of Lower Saxony (Nidersächsisches Landesamt für Verbraucherschutz und Lebensmittelsicherheit, LAVES, Lower Saxony, Germany) with number TVA 15/1890.

### Bleomycin-Induced Lung Fibrosis

Lung fibrosis was induced by intratracheal instillation of a single dose of BLM (4U/kg) (Sigma, USA) dissolved in 200 μl of sterile saline under isoflurane anesthesia. Control animals received the same volume of saline. The animal body weights were recorded every day during the course of the experiment.

### Isolation of Alveolar Epithelial Type II Cells

Fresh alveolar epithelial type II cells (AE2C) were isolated from healthy donor animals. The protocol for purification has been described by Richards RJ group (26). Briefly, to isolate AE2C, the lungs were removed from the animal and lavaged with 5 × 10 ml saline. The lungs were digested by filling with 0.25% trypsin dissolved in saline (100 ml) (T8003, Sigma, Missouri, USA) and suspended in 0.9% NaCl at 37°C for 30 min, with the trypsin constantly topped up to expand the parenchyma for 30 min, suspended in a saline solution at 37°C. Following digestion, the lungs were chopped into 1–2 mm^2^ cubes, treated with 75 U/ ml DNase dissolved in saline and filtered through nylon meshes ranging from 150 to 30 μm. The resulting cell suspension was centrifuged (250 × *g*, 20 min at 10°C) through a sterile Percoll gradient and the AE2C rich band was removed. A second DNase treatment of 20 U/ml was administered, and the cells were recovered as a pellet by centrifugation at 250 × *g* for 20 min. These cells were resuspended in 5 ml DCCM 1 (Biological Industries, Kibbutz Beit Haemek, Israel) completed with a 2% (w/v) L- Glutamine and subjected to differential attachment on a plastic Petri dish. Non-adherent AE2C were collected after 2 h and counted to establish the final cell yield of freshly purified cells.

The AE2C viability was assessed by Trypan Blue (Sigma, Missouri, USA), showing >95% viability. Cell yield, purity and characterization of freshly isolated AE2C were established by the presence of intracellular alkaline phosphatase (Sigma, USA).

### Transplantation Procedure

At day 3 or 7 after intratracheal BLM, recipient animals were transplanted with AE2C by intratracheal instillation (2.5 × 10^6^ cells/animal suspended in 400 μl of sterile saline) under isoflurane anesthesia. The control group received the same dose of cells 3 or 7 days after saline instillation. The animals were sacrificed at day 7 and 14 after the induction of lung fibrosis to plot time dependent changes in lung physiology and functionality.

### Experimental Groups

**Figure 1:**
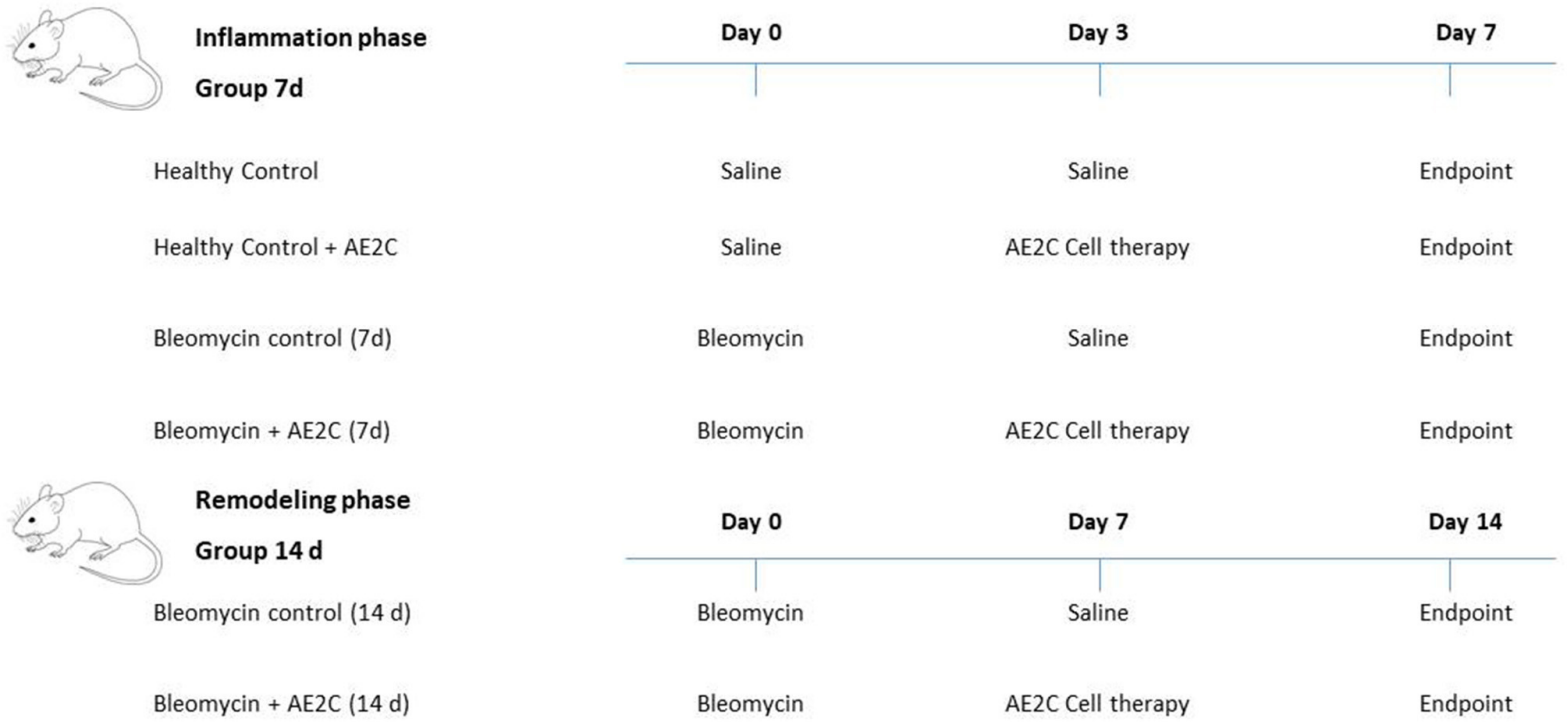
Scheme of the experimental design. The animals were randomly distributed into four experimental groups and two different time points during the inflammatory phase (Group 7d) and fibrotic phase (Group 14 d) (*n* = 5–8 in each group).

The animals were randomly distributed into four experimental groups and we studied two different time points (*n* = 5–8 in each group): Healthy Control: Saline instillation; Healthy Control + AE2C (3 days after saline instillation); Bleomycin control: Bleomycin instillation + saline (3 or 7 days after bleomycin instillation); Bleomycin + AE2C: Bleomycin instillation + alveolar type II cell transplantation (3 or 7 days after bleomycin instillation). Figure 1 shows a scheme of the experimental design.

### Invasive Pulmonary Function Test

To assess the pressures and rates at which alveoli in the injured lungs closed, de-recruitability tests at different PEEP (positive end-expiratory pressures) as well as quasi-static pressure volume-loops using a Flexivent ventilator (SCIREQ, Canada) were performed (27). The de-recruitability tests consist of two recruitment maneuvers (up to 30 cmH_2_O followed) followed by 5 min of low-tidal volume ventilation (10 ml/kg body weight) interspersed with 8 s multi-frequency forced oscillation perturbations at 30 s intervals. Tissue elastance (H), tissue damping (G) and tissue hysteresivity (G/H) were calculated by fitting the constant phase model to impedance spectra. After each de-recruitability test, 3 quasi-static PV loops were recorded, and quasi-static compliance was calculated according to the Salazar-Knowles equation.

### Perfusion-Fixation of Lungs and Sampling Procedures for Light and Electron Microscopy

Fixation and sampling were conducted according to standards on quantitative morphology of the lung (28). The lungs were fixed by vascular perfusion at an airway pressure of 13 cm H_2_O on expiration. The volume of the lungs was determined and followed by a systematic uniform random sampling. Seven to 9 tissue slices per lung were randomized for light microscopy and 5–6 tissue blocks per lung for electron microscopic evaluation. Slices for light microscopy were embedded in Technovite resin and stained with toluidine blue. Lung blocks for electron microscopy were embedded in Epon resin and contrasted with uranyl acetate and lead citrate.

### Design-Based Stereology

At the light microscopic level, a newCAST-system (Visiopharm A/S, Denmark) was used to perform systematic uniform random area sampling and to superimpose an appropriate test system on the fields of view. A transmission electron microscope (FEI Morgagni, Netherlands) equipped with a digital camera (Olympus Soft Imaging Systems, Germany) was used to obtain representative fields of view. The parameters useful to characterize the pathology of fibrotic development were volume of lungs, volume of parenchyma, volume of ventilated parenchyma, thickness of the septal wall, volume fraction of AE2C cells, volume of lamellar bodies per AE2C cell, volume of intra-alveolar surfactant, lamellar body like structures, multilamellar vesicles, tubular myelin, and unilamellar vesicles. A stereology tool (STEPanizer®, Bern, Switzerland) was employed for definitive stereological morphometry. A point grid was chosen as a test system for volume estimation. Securing sufficient stereological precision, the number of test points was adjusted to a minimum of 200 to 300 counting events per parameter per lung (29). A counting event was defined as a match of a structure of interest (SOI) with the test probe. At 5x magnification, volume fractions of parenchyma [Vv(par/lung)] and non-parenchyma [Vv(non-par/lung)] were obtained. Parenchyma was defined as lung tissue enabling gas exchange, comprising septa and airspaces, and was differentiated in ventilated [Vv(ventpar/par)] and non-ventilated parenchyma [Vv(non-vent/par)]. Pleura, conducting airways and large vessels with the surrounding connective tissue were defined as non-parenchyma. Volume densities of ductal airspaces [Vv(duct/par), alveolar airspaces (Vv(alv/par) and alveolar septa (Vv(sept/par)] were determined at 20x magnification within ventilated parenchyma. Herein, an additional test system consisting of 4 line-pairs was utilized for counting intersections of test probes and alveolar surface. All analyzed parameters for lung structure regarding fibrosis were chosen according to recommendations from Ochs and Mühlfeld (29) for stereology in pulmonary fibrosis and (30) for stereology in bleomycin induced lung injury and fibrosis.

Volume fractions [e.g., Vv(par/lung)] were calculated by dividing the number of points (P) hitting the SOI (structure of interest) by the number of points hitting the reference space, e.g., total lung.

$$\begin{array}{l} \text{Vv}\left(\text{par}/\text{lung}\right)=\sum \left[\text{Ppar}\right]/\sum \left[\text{Ppar}+\text{Pnon}-\text{par}\right] \end{array}$$

Multiplication of the volume fraction with total lung volume provided total volumes of each SOI [e.g., V(par,lung)]: V(par,lung) = Vv(par/lung)^*^V(lung),

Analogous calculations were performed for parenchymal components, e.g., alveolar airspaces, LB in AE2C and TM fraction in total intra-alveolar surfactant:

$$\begin{array}{l} \text{Vv}\left(\text{alv}/\text{ven}-\text{par}\right)=\sum \left[\text{Palv}\right]/\sum \left[\text{Pvent}-\text{par}\right]\\ \text{ V}\left(\text{alv},\text{ vent}-\text{par}\right)=\text{Vv}{\left(\text{alv}/\text{vent}-\text{par}\right)}^{*}\text{V}\left(\text{vent}-\text{par},\text{ lung}\right), \end{array}$$

Intersection (I) countings were utilized in combination with the length per point of the test system (l(p)) to estimate the alveolar surface density of ventilated parenchyma. The calculated absolute volume describes the alveolar surface contributing to pulmonary gas exchange:

$$\begin{array}{l} \text{Sv}\left(\text{alv}/\text{vent}-\text{par}\right)=\left(2^{*}\sum \left[\text{I}\right]\right)/(\text{l}{\left(\text{p}\right)}^{*}\sum \left[\text{Pvent}-\text{par}\right]\\ \text{ Salv}=\text{ Sv}{\left(\text{alv}/\left(\text{vent}-\text{par}\right)\right)}^{*}\text{V}\left(\text{vent}-\text{par},\text{ lung}\right), \end{array}$$

Septal thickness was computed as follows: τ (sep) = Vv(sept/par)/Sv(alv/par)^*^2.

### Statistical Analysis

Data are expressed as mean values for each subject, horizontal bars represent the mean of the group. In bar graphs, data is represented as mean and SD in error bars. Statistical analysis was carried out by a non-parametric analysis (Kruskal-Wallis test) followed by appropriate *post hoc* tests, Dunn's multiple comparisons test when differences were significant (GraphPad Software Inc, USA). A *p* < 0.05 was considered significant.

## Results

### Alveolar Dynamics of Bleomycin Induced Lung Injury (d7) and Fibrosis (d14) After AE2C Transplantation

**Figure 2:**
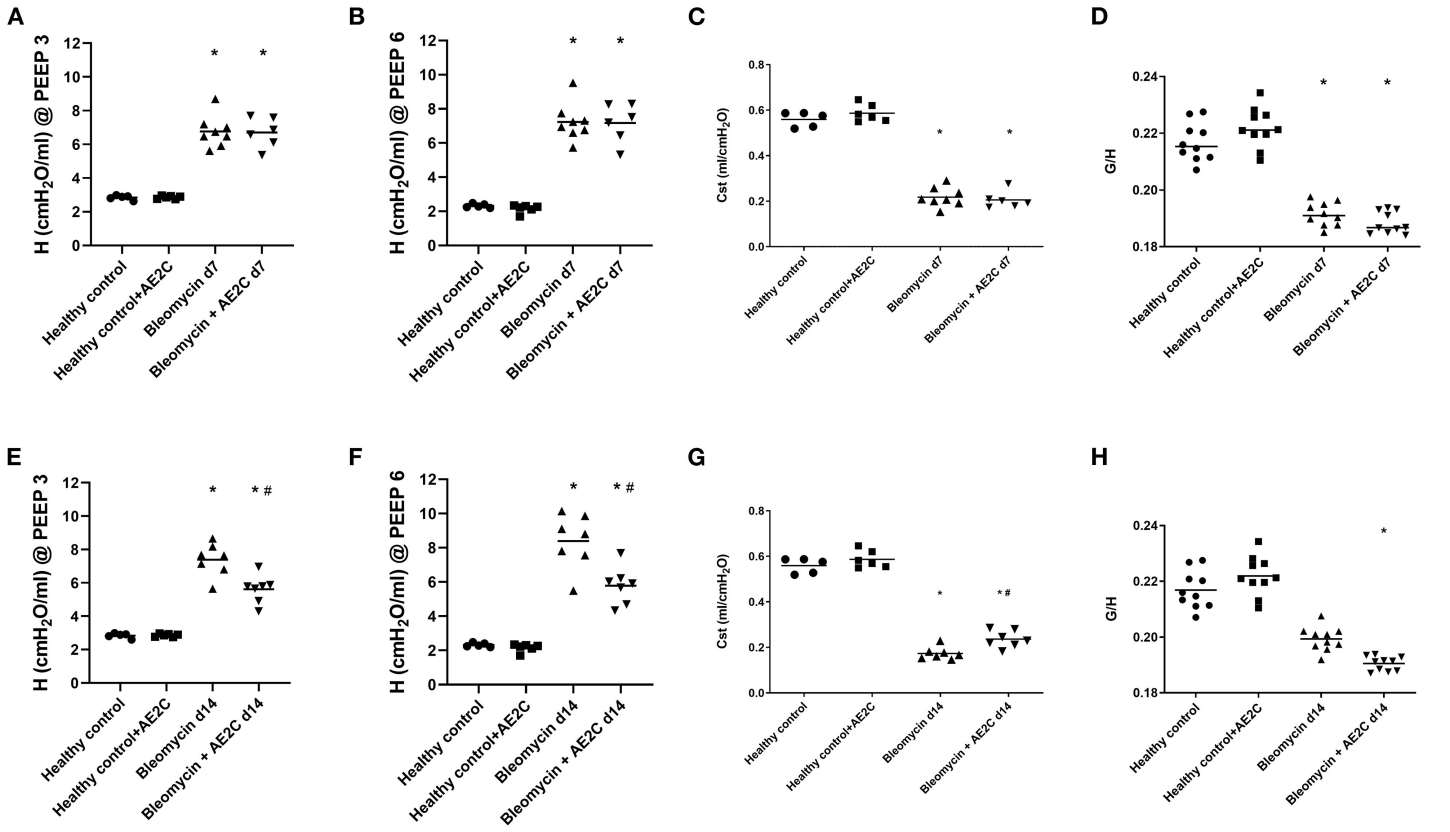
Alveolar dynamics of bleomycin induced lung injury (d7) and fibrosis (d14) after AE2C transplantation. **(A–D)** Alveolar dynamics of bleomycin induced lung injury (7 days after bleomycin application) and AE2C transplantation (3 days after bleomycin application). **(A)** Tissue elastance (H) at a positive end-expiratory pressure (PEEP) of 3cmH2O over the 5 min forced oscillation technique (FOT) at day 7 after bleomycin application. **(B)** Tissue elastance (H) at a PEEP of 6cmH2O at day 7 after bleomycin application. **(C)** Static compliance (ml/cmH2O) at day 7 after bleomycin application. **(D)** Tissue histeresivity **(G,H)** at day 7 after bleomycin application. **(E–H)** Alveolar dynamics of bleomycin induced lung fibrosis (14 days after bleomycin application) and AE2C transplantation (7 days after bleomycin application). **(E)** Tissue elastance **(H)** at a positive end-expiratory pressure (PEEP) of 3cmH2O over the 5 min forced oscillation technique (FOT) at day 14 after bleomycin application. **(F)** Tissue elastance **(H)** at a PEEP of 6cmH2O at day 14 after bleomycin application. **(G)** Static compliance (ml/cmH2O) at day 14 after bleomycin application. **(H)** Tissue histeresivity **(G,H)** at day 14 after bleomycin application. *p < 0.05 vs. healthy controls, #*p* < 0.05 vs. bleomycin control.

After isolation of healthy AE2C, the purity of the cells measured by positive staining with alkaline phosphatase was 87 ± 2%. After transplantation of AE2C, we performed a complete alveolar dynamics analysis by means of Forced Oscillation Technique (FOT) in a small animal ventilator. Figures 2A,B shows the elastance (H) of the lungs 7 days after bleomycin application and 4 days after AE2C transplantation in the corresponding treatment and control groups. As already described, elastance is significantly increased after bleomycin application at both PEEP of 3 (Figure 2A) and 6 cmH2O (Figure 2B) compared to healthy controls. In addition, after AE2C transplantation, no changes in elastance could be observed compared to bleomycin control. In accordance, the application of bleomycin reduced static compliance (Figure 2C) and AE2C showed no improvement of this value. Tissue hysteresivity (G/H) was also significantly reduced in the groups with application of bleomycin with no changes after AE2C transplantation (Figure 2D).

When analyzing the alveolar dynamics 14 days after bleomycin application, and 7 days after AE2C transplantation, an improvement on elastance (Figures 2E,F) and compliance (Figure 2G) could be observed. The group that received an AE2C transplantation after bleomycin application showed significantly lower elastance values than the bleomycin control group, between those of the bleomycin control group and the healthy groups. This could be related to a beneficial effect of the newly transplanted AE2C in the mechanical properties of the lung. Accordingly, static compliance was also significantly higher in the disease group that received the AE2C transplantation than the bleomycin control group. Tissue hysteresivity was also affected by AE2C transplantation, showing a significant reduction (Figure 2H).

### Lung Structure of Bleomycin Induced Lung Injury (d7) and Fibrosis (d14) After AE2C Transplantation

**Figure 3:**
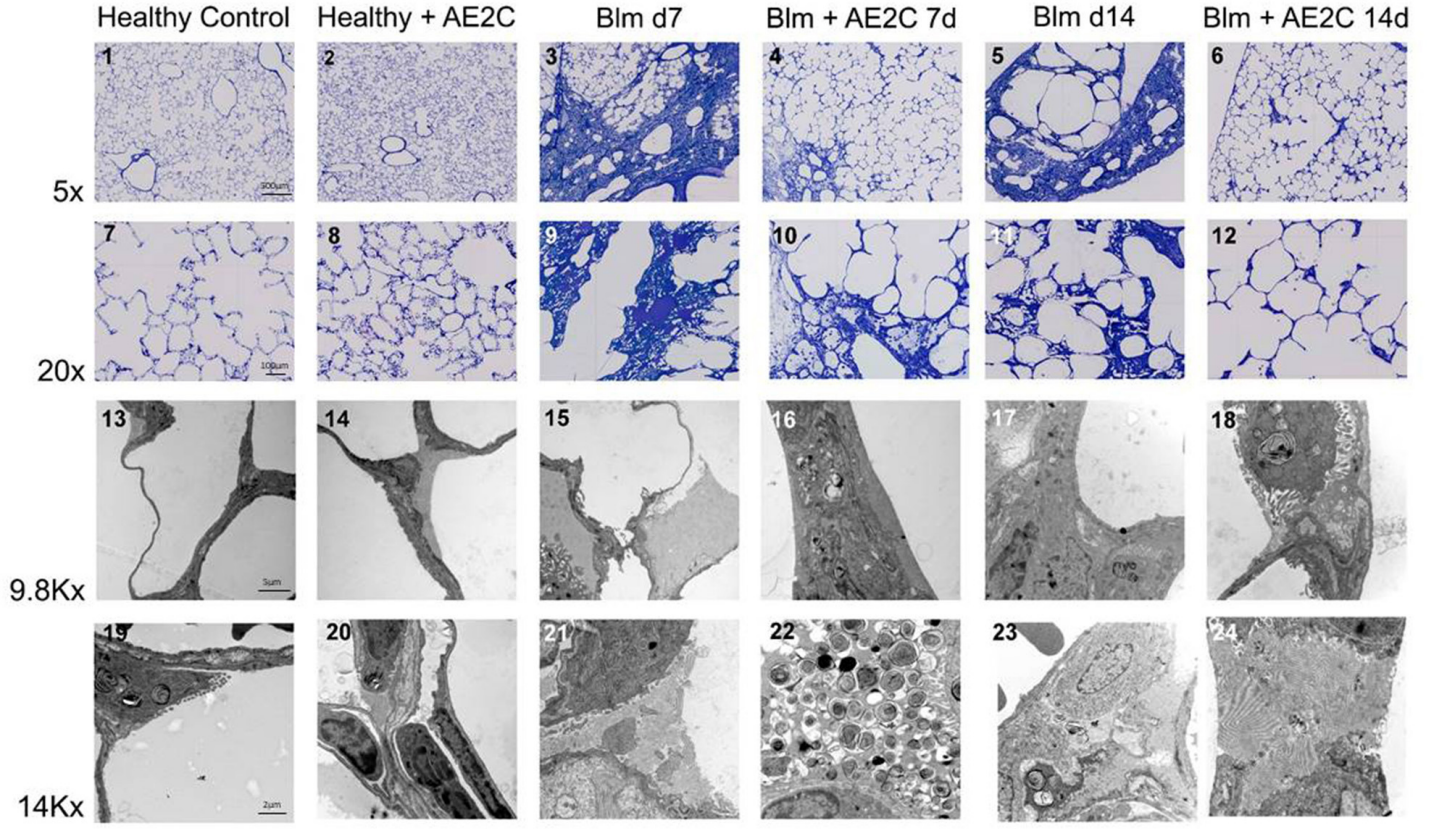
Representative light and electron microscopy pictures of bleomycin induced lung injury (d7) and fibrosis (d14) after AE2C transplantation. Upper panels (micrographs 1-12): toluidine blue stained tissue at 5 and 20X magnification of the different experimental groups. Bottom panels (micrographs 13-24): electron microscopy pictures at 9.8 and 14Kx magnification of the different experimental groups.

**Figure 4:**
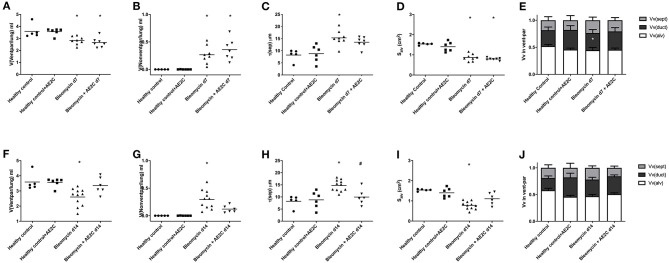
Lung structure of bleomycin induced lung injury (d7) and fibrosis (d14) after AE2C transplantation. **(A–E)** Lung structure of bleomycin induced lung injury (7 days after bleomycin application) and AE2C transplantation (3 days after bleomycin application). **(A)** Total volume of ventilated parenchyma per lung (ml) at day 7 after bleomycin application. **(B)** Total volume of non-ventilated parenchyma per lung (ml) at day 7 after bleomycin application. **(C)** Thickness of septal wall (τ) at day 7 after bleomycin application. **(D)** Total alveolar surface per lung (S_alv_). **(E)** Volume fractions of alveolar spaces (white bar), ductal spaces (dark gray bar) and septal wall (light gray bar) per ventilated parenchyma at day 7 after bleomycin application. **(F–J)** Lung structure of bleomycin induced lung fibrosis (14 days after bleomycin application) and AE2C transplantation (7 days after bleomycin application). **(F)** Total volume of ventilated parenchyma per lung (ml) at day 14 after bleomycin application. **(G)** Total volume of non-ventilated parenchyma per lung (ml) at day 14 after bleomycin application. **(H)** Thickness of septal wall (τ) at day 14 after bleomycin application. **(I)** Total alveolar surface per lung (S_alv_). **(J)** Volume fractions of alveolar spaces (white bar), ductal spaces (dark gray bar) and septal wall (light gray bar) per ventilated parenchyma at day 14 after bleomycin application. *p < 0.05 vs. healthy controls, #*p* < 0.05 vs. bleomycin control.

In order to understand if the mechanical parameters are a reflection of changes in lung structure, we immediately inflated and fixed the lungs of the animals after performing the alveolar dynamics in the small animal mechanical ventilator. Figure 3 shows representative micrographs of the lungs from the animals at light (two upper panels, micrographs 1–12) and electron microscopy (two bottom panels, micrographs 13–24) level to illustrate the quantitative results shown in the following figures. As expected from stiffer and less compliant injured lungs (d7), the lung volume measured by fluid displacement and the volume of air used for inflation at constant pressure, is reduced in the bleomycin groups with and without AE2C transplantation (Supplementary Figures 1A,B). Looking closely at the parenchymal tissue, we also observed a significant decrease in ventilated (Figure 4A) and an increase of non-ventilated (Figure 4B) parenchyma total volume (Figure 3, micrographs 3 and 9). In addition, we also observed a significant increase in septal wall thickness (Figure 4C) and a decrease in total alveolar surface (Figure 4D) in the bleomycin treated with and without AE2C transplantation (Figure 3, micrographs 3-4 and 9-10). Within ventilated parenchyma, the alveolar spaces seemed to be the most affected by bleomycin application (Figure 4E and Supplementary Figures 2A–C). While there was a trend to increase in ductal volume density (Figure 4E), the total volume of ductal spaces remained unchanged (Supplementary Figure 2B), leading us to think that the air lost in the alveolar side is due to collapse and is not over-distending alveolar ducts.

On the other hand, the AE2C transplantation in lungs undergoing fibrotic remodeling showed more promising results (Figure 3, micrographs 5-6 and 11-12). According to the mechanical parameters presented above, the decrease in elastance and the increase in compliance in the transplanted group compared to the bleomycin group, was accompanied by an increase in ventilated parenchyma (Figure 4F) and a decrease in non-ventilated parenchyma (Figure 4G). In addition, there was a significant reduction of the septal wall thickness (Figure 4H) and increased alveolar surface (Figure 4I). As for the treatment during lung injury, within ventilated parenchyma, the alveolar spaces seemed to be the most affected ones by the bleomycin application and the transplantation treatment (Figure 4J and Supplementary Figures 2D–F). In this case, a significant increase in total volume of alveolar spaces (Supplementary Figure 2D) was observed in the bleomycin treated and transplanted lung compared to the bleomycin control.

Taking all together, the improved mechanics shown by the fibrotic lung treated with AE2C seemed to be supported by an improved lung structure by means of increased opened alveolar spaces and surface with thinner septal walls.

### Lung Surfactant Ultrastructure of Bleomycin Induced Lung Injury (d7) and Fibrosis (d14) After AE2C Transplantation

In order to dissect the fine structure and composition of the alveolar parenchyma, we further analyzed the lung ultrastructure by means of quantification using electron microscopy micrographs (Figure 3, micrographs 13-24). This analysis allowed us to look closely at AE2C total volume in the alveolar parenchyma, as well as the total volume of edema and extracellular matrix. In addition, we have quantified the volume fraction of lamellar bodies (LB) in AE2C, to understand if surfactant synthesis is influenced by the AE2C transplantation.

**Figure 5:**
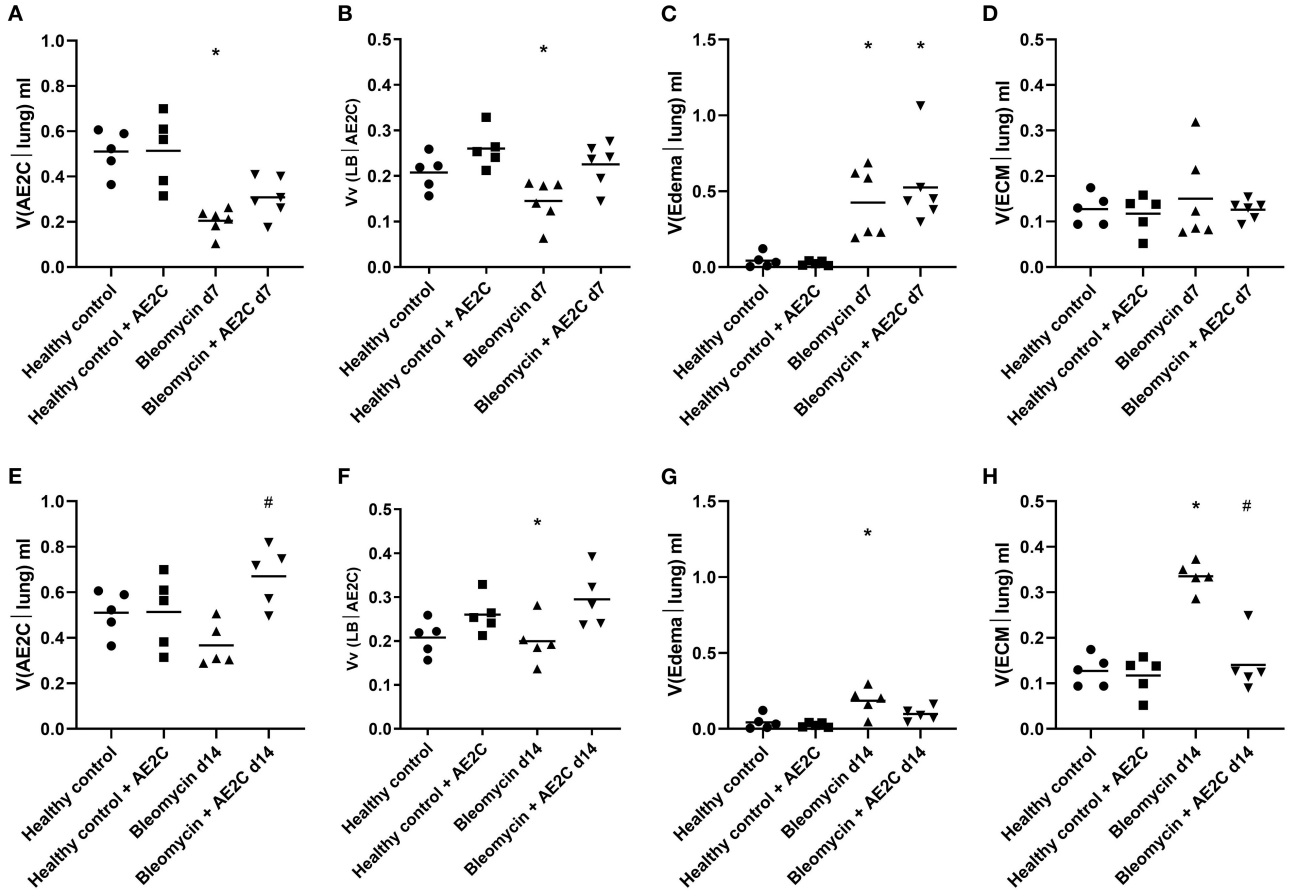
Lung ultrastructure of bleomycin induced lung injury (d7) and fibrosis (d14) after AE2C transplantation. **(A–D)** Lung ultrastructure of bleomycin induced lung injury (7 days after bleomycin application) and AE2C transplantation (3 days after bleomycin application). **(A)** Total volume of AE2C in alveolar parenchyma at day 7 after bleomycin application. **(B)** Volume fraction of lamellar bodies (LB) in AE2C at day 7 after bleomycin application. **(C)** Total volume of edema in alveolar parenchyma at day 7 after bleomycin application. **(D)** Total volume of extracellular matrix (ECM) in alveolar parenchyma at day 7 after bleomycin application. **(E–H)** Lung ultrastructure of bleomycin induced lung injury (14 days after bleomycin application) and AE2C transplantation (7 days after bleomycin application). **(E)** Total volume of AE2C in alveolar parenchyma at day 14 after bleomycin application. **(F)** Volume fraction of LB in AE2C at day 14 after bleomycin application. **(G)** Total volume of edema in alveolar parenchyma at day 14 after bleomycin application. **(H)** Total volume of ECM in alveolar parenchyma at day 14 after bleomycin application. *p < 0.05 vs. healthy controls, #*p* < 0.05 vs. bleomycin control.

As described before (30), the application of bleomycin reduces the volume of AE2C and the transplantation of AE2C slightly changed this (Figure 5A). However, the volume fraction of LB inside AE2C showed a trend to increase (non-statistically significant) in the transplanted injured group compared to the bleomycin group (Figure 5B). Also according to previous reports, bleomycin induced the formation of alveolar edema and its volume was unchanged after transplantation (Figure 3, micrograph 15 and 21, Figure 5C). Since the volume of ECM was not changed in any group (Figure 5D), the increase in septal wall thickness reported above (Figure 4C) is likely due to the alveolar edema and not to a remodeling process in the ECM.

When looking at fibrotic lungs (d14), the decrease in volume of AE2C after bleomycin was not significant compared to control groups, but the AE2C transplantation resulted in an increased total volume of these cells over the values of the controls (Figure 5E). The volume fraction of LB in AE2C also showed the same pattern, non-statistically significant against the bleomycin control (Figure 5F). Even when the volume of edema in bleomycin treated lungs was still significantly higher than in controls (Figure 5G), the values are much lower than the lung injured (d7) groups (Figure 5C). However, the volume of ECM was notably increased in the bleomycin group 14 days after application, and significantly reduced after transplantation (Figure 5H). Therefore, the increase in septal wall thickness described above (Figure 4H) could be mainly related to aberrant accumulation of ECM components.

As AE2C are responsible for surfactant synthesis and secretion, we investigated whether the volume fraction of surfactant in alveolar spaces was affected by the AE2C transplantation. In addition, we were also interested in describing if the surfactant present in the alveolar spaces presented different fractions of active (tubular myelin (TM), lamellar body-like (LBL) and multilamellar vesicles (MLV) or inactive (unilamellar vesicles (ULV)) forms (9, 31) within the different disease stages or treatments.

**Figure 6:**
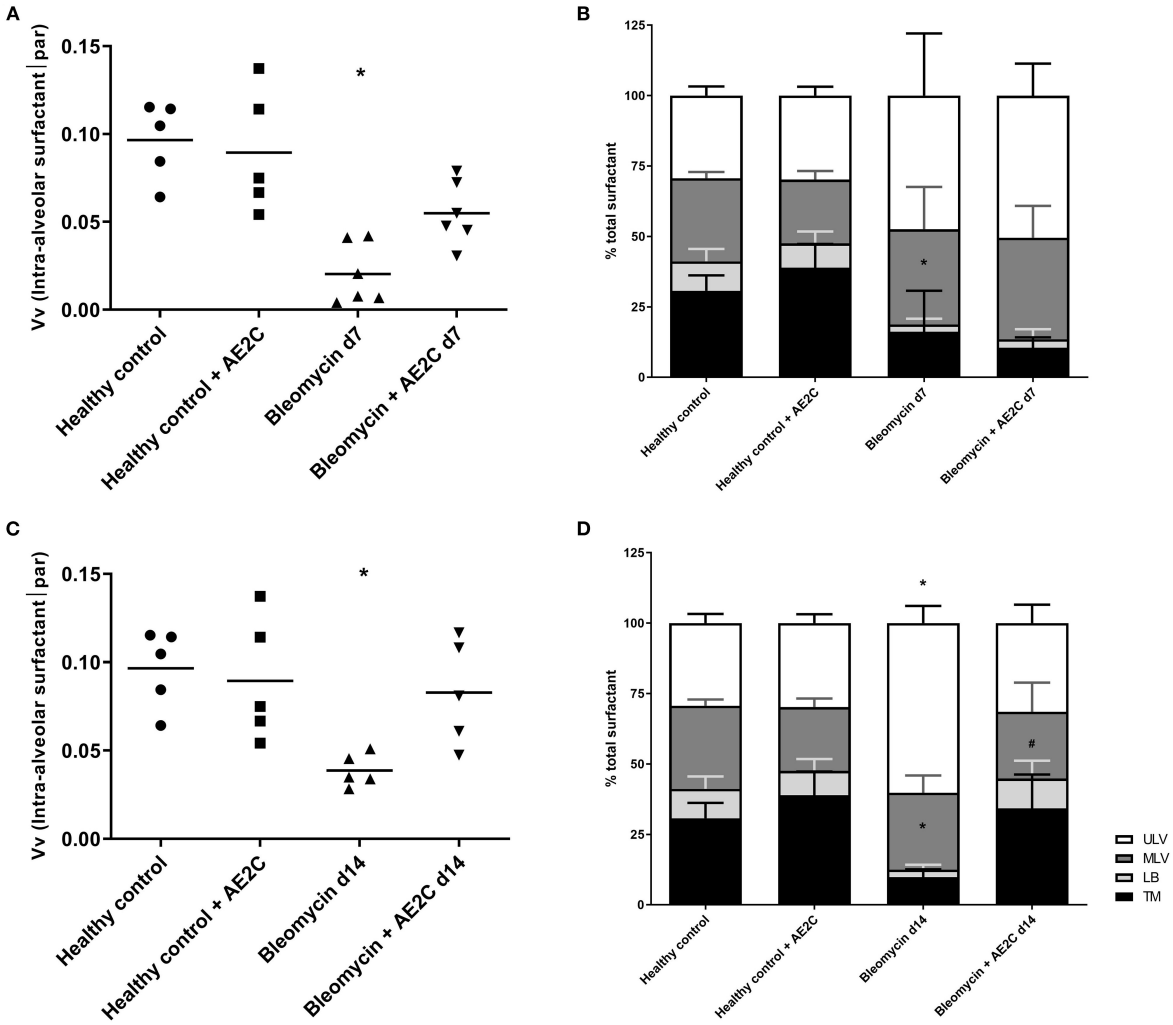
Lung surfactant ultrastructure of bleomycin induced lung injury (d7) and fibrosis (d14) after AE2C transplantation. **(A,B)** surfactant ultrastructure of bleomycin induced lung injury (7 days after bleomycin application) and AE2C transplantation (3 days after bleomycin application). **(A)** Volume fraction of intra-alveolar surfactant in alveolar parenchyma at day 7 after bleomycin application. **(B)** Proportion of different surfactant ultrastructures, tubular myelin (TM, black bars), lamellar body-like (LBL, light gray bars), multilamellar vesicles (MLV, dark gray bars) and unilamellar vesicles (ULV, white bars) at day 7 after bleomycin application. **(C,D)** Surfactant ultrastructure of bleomycin induced lung fibrosis (14 days after bleomycin application) and AE2C transplantation (7 days after bleomycin application). **(A)** Volume fraction of intra-alveolar surfactant in alveolar parenchyma at day 14 after bleomycin application. **(B)** Proportion of different surfactant ultrastructures, tubular myelin (TM, black bars), lamellar body-like (LBL, light gray bars), multilamellar vesicles (MLV, dark gray bars) and unilamellar vesicles (ULV, white bars) at day 14 after bleomycin application. *p < 0.05 vs. healthy controls, #*p* < 0.05 vs. bleomycin control.

For both experimental settings, the application of bleomycin significantly reduced the volume fraction of intra-alveolar surfactant and its active forms (Figure 6). AE2C transplantation during lung injury resulted in a non-significant increase of volume fraction of intra-alveolar surfactant (Figure 6A) compared to the bleomycin group. In addition, the transplantation did not change the reduction of TM and LBL induced by bleomycin, and inversely the increase on ULV (Figure 6B). On the other hand, AE2C transplantation in the lungs undergoing fibrotic remodeling showed a promising, but not significant, increase of volume fraction of intra-alveolar surfactant, accompanied by a statistically significant increase in TM and LBL active forms of surfactant (Figure 3, micrograph 24, Figures 6C,D).

### Lung Structure-Mechanics Relationships of Bleomycin Induced Lung Injury (d7) and Fibrosis (d14) After AE2C Transplantation

**Figure 7:**
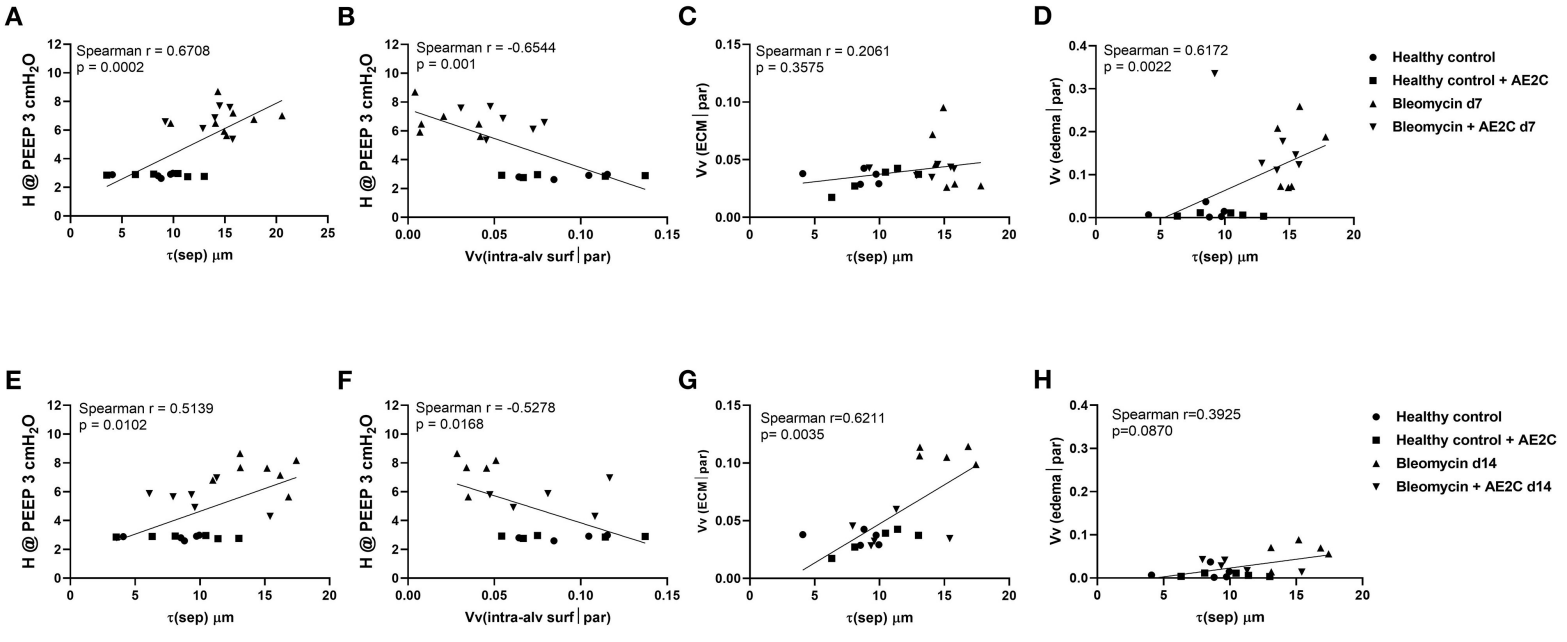
Alveolar dynamics and structure correlations in bleomycin induced lung injury (d7) and fibrosis (d14) after AE2C transplantation. **(A–D)** Alveolar dynamics and structure correlation of bleomycin induced lung injury (7 days after bleomycin application) and AE2C transplantation (3 days after bleomycin application). **(A)** Correlation between tissue elastance at PEEP 3 cmH2O and septal wall thickness (τ) at day 7 after bleomycin application. **(B)** Correlation between tissue elastance at PEEP 3 cmH2O and volume fraction of intra-alveolar surfactant in alveolar parenchyma at day 7 after bleomycin application. **(C)** Correlation between volume fraction of ECM and septal wall thickness at day 7 after bleomycin application. **(D)** Correlation between alveolar edema and septal wall thickness at day 7 after bleomycin application. **(E–H)** Alveolar dynamics and structure correlation of bleomycin induced lung injury (14 days after bleomycin application) and AE2C transplantation (7 days after bleomycin application). **(A)** Correlation between tissue elastance at PEEP 3cmH2O and septal wall thickness (τ) at day 14 after bleomycin application. **(B)** Correlation between tissue elastance at PEEP 3cmH2O and volume fraction of intra-alveolar surfactant in alveolar parenchyma at day 14 after bleomycin application. **(C)** Correlation between volume fraction of ECM and septal wall thickness at day 14 after bleomycin application. **(D)** Correlation between alveolar edema and septal wall thickness at day 14 after bleomycin application. *p < 0.05 vs. healthy controls, #*p* < 0.05 vs. bleomycin control.

Lastly, in order to investigate which structural parameters may impact the mechanical properties of the lungs after the different treatments and potential causal relationships, we systematically correlated the mechanical and structural data. In Figure 7, the most interesting correlations are presented for either the bleomycin induced lung injury (Figures 7A–D) and fibrosis (Figures 7E–H) after transplantation. Even though we found a significant correlation between tissue elastance and thickness of the septal wall (Figure 7A) and elastance and volume fraction of intra-alveolar surfactant (Figure 7B), there is no effect of the therapy in the bleomycin induced lung injury model (d7). In addition, the increase in septal wall thickness previously described here is not significantly correlated with the total volume of extracellular matrix (Figure 7C), but with the volume fraction of edema (Figure 7D). Therefore, it seems that edema is the main structural change induced in this model and preventing the transplantation treatment to have a positive effect. On the other hand, when the treatment is performed during the fibrotic remodeling phase, the correlation of tissue elastance and septal wall thickness (Figure 7E) and tissue elastance and volume fraction of intra-alveolar surfactant (Figure 7F) were statistically significant and showed the transplanted group to be between the control and disease groups. In addition, in this experimental setting, the main structural component change in the septal walls was the increase in total volume of extracellular matrix (Figure 7G). Edema was not statistically significantly correlated to septal wall thickness (Figure 7H). Therefore, remodeling and aberrant accumulation of extracellular matrix seemed to be the main component of the increase of septal wall thickness. Very interestingly, the transplantation with AE2C seemed to prevent this aberrant accumulation of extracellular matrix and consequently the septal wall thickness was not increased.

## Discussion

Damage/injury and apoptosis of AE2C (32–34) is a well-described process contributing to lung remodeling. AE2C are essential cells for the proper functioning of the lung and surfactant homeostasis (8, 35). Therefore, a therapeutic strategy to replace the injured AE2C may help to improve the disease outcome. Previously, Serrano-Mollar et al. demonstrated, in preclinical studies, that AE2C intratracheal transplantation was able to reduce fibrosis and restore pulmonary surfactant proteins levels (18, 19). They also observed that the induced pluripotent stem cells (iPSCs) derived AE2C reduce fibrosis by inhibiting TGF-β and α-SMA expression (23). Furthermore, in a clinical study performed with IPF patients, the intratracheal administration of heterologous AE2C was safe, well-tolerated and with no relevant side effects. Furthermore, this cell therapy was able to stabilize disease progression and improve health-related quality of life throughout a 1-year clinical follow-up (25). The functionality tests evaluated in that clinical study were DLCo and FVC (25), although these results provide information regarding lung capacity and performance, to date it has not been assessed whether these changes involve any improvement in lung structure functionality. Here, we studied the mechanical and structural changes induced by bleomycin and changed by the AE2C transplantation in order to contribute to understanding the effect of the replacement of injured AE2C as a therapeutic approach. Interestingly, we can conclude that when performing the treatment early during the lung injury phase of the bleomycin model, mainly edema and edematous material seem to impose a barrier to the potential beneficial effect of the transplanted cells. As already described (36), the formation of alveolar edema is one of the earliest events induced by the intratracheal application of bleomycin. Lutz and colleagues (30) also showed that already at day 3 after bleomycin application, the presence of edema and inactivation of surfactant, showing abnormally high surface tension, lead to a decreased number of opened alveoli. Accordingly, we found that 7 days after bleomycin application, the volume of ventilated parenchyma (Figure 4A) as well as total alveolar surface (Figure 4D) was reduced. In addition, there was an increase in septal wall thickness, mainly due to the formation of alveolar edema (Figures 4C, 5C, 7D), rather than a remodeling process with ECM accumulation (Figure 7C).

We found a non-statistical significant increase in the volume fraction of intra-alveolar surfactant in the transplanted group compared to the bleomycin diseased group (Figure 6A) in accordance with the increased volume fraction of LB in AE2C (Figure 5B). However, when analyzing the ultrastructure of this surfactant, we found an increased amount of ULV, as in the bleomycin control group, which seems to be the result of an inactivating process of surfactant by the edematous material, as previously reported (37–41). As surfactant remained inactivated, and edema was still present, we could not observe any improvement in alveolar dynamics. Therefore, freshly isolated and transplanted AE2C could not help in resolving edema and/or activating/replacing surfactant.

On the other hand, when transplanting the AE2C during the fibrotic remodeling phase, we can observe a promising beneficial effect of the therapy in improving alveolar dynamics and lung structure. At day 14 after bleomycin application, tissue elastance is further increased (Figures 2E,F) as a consequence of a statistically significant reduced volume of ventilated parenchyma and total alveolar surface, with the associated increase in non-ventilated parenchyma and septal wall thickness (Figures 4A–I). Interestingly, at this stage the total volume of edema is minimally increased (Figure 5G) compared to d7 (Figure 5C), whereas the volume of ECM is statistically significantly increased (Figure 5H), as previously described (30, 36). Therefore, in this case, the increase in septal wall thickness may be mainly due to the aberrant accumulation of ECM (Figure 7G), rather than alveolar edema (Figure 7H). In addition, bleomycin application also impacted the volume fraction of intra-alveolar surfactant (Figure 6C) and induced the conversion of active to inactive structures of lung surfactant (Figure 6D), as previously shown (30). The transplantation of AE2C at day 7, prevented the accumulation of ECM (Figure 5H), and therefore the increase in septal wall thickness (Figure 4H), resulting in a statistically significant reduction of elastance and an increase in compliance in alveolar dynamics (Figures 2E–H). A higher PEEP during forced oscillation perturbation was linked with a reduced tissue elastance in healthy controls but an increase in untreated bleomycin challenged lungs at day 14. The AE2C transplantation could convert this PEEP-dependent behavior of tissue elastance to normal – a higher PEEP resulted in a reduction in tissue elastance. In healthy lungs, the increase in the PEEP level from 3 to 6 cmH_2_O results in a recruitment of folds in the alveolar walls and probably also alveolar shape changes without overdistension which would place ventilated airspaces to the upper non-linear portion of their pressure volume relationship. In bleomycin challenged lungs with their heterogeneous ventilation due to higher fraction of non-ventilated lung parenchyma and thickened septa those distal airspaces which are still ventilated become overstretched between PEEP 3 and 6 cmH_2_O so that tissue elastance increases. Such an overdistension of lung parenchyma which occurs already at quite low airway opening pressures might represent an additional trigger for fibrotic remodeling even during spontaneous breathing e.g., by release of active TGF-β1 as demonstrated by (14). AE2C transplantation at day 7 results in a more homogenous ventilation of the lung as indicated by the reduced fraction of non-ventilated lung parenchyma, reduced septal wall thickness and improved alveolar surface area so that the reduction in tissue elastance at PEEP 6 compared to PEEP 3 cmH_2_O can be interpreted by recruitment of folding or even complete alveoli without overdistension of distal airspaces so that at the organ scale the pressure-volume relationship does not reach the upper non-linear portion. Hence, in this range of airway opening pressures there is no hint for overdistension and therefore pro-fibrotic mechanical stress. The improvement in homogeneity of ventilation within the lung after AE2C transplantation might be a consequence of improved regeneration and/or reduction in surface tension.

Of note, the transplantation of freshly isolated healthy AE2C at day 7, induced the increase in total volume of AE2C and volume fraction of LB in AE2C, which persisted 7 days later (day 14) compared to the bleomycin control group. This seems to be related to the secretion of functional surfactant, as the volume fraction of intra-alveolar surfactant recovered to healthy levels (Figure 6C) and the most active structures of surfactant (mainly TM and LBL) were also similar to the controls (Figure 6D). Therefore, we can conclude that the transplantation of AE2C showed to be very effective in recovering the healthy status of lung surfactant, contributing to prevent ECM accumulation and septal wall thickening, resulting in softer lung tissue.

Taking all together, even though the treatment with AE2C shows no effect in the treatment of lung injury, this treatment is able to prevent the main features of lung fibrotic remodeling, mainly aberrant accumulation of ECM and thickening of the septal wall. Whether this effect is a direct consequence to the newly secreted active surfactant deserves further research. However, it has already been described that mechanical stress, for example, due to surfactant dysfunction and increased surface tension, may contribute to the fibrotic remodeling (42). Preventing this mechanical stress by the production and secretion of active surfactant may be one of the mechanisms of action in this therapeutic model.

## Data Availability Statement

The raw data supporting the conclusions of this article will be made available by the authors, without undue reservation.

## Ethics Statement

The animal study was reviewed and approved by Lower Saxony (Nidersächsisches Landesamt für Verbraucherschutz und Lebensmittelsicherheit, LAVES, Lower Saxony, Germany) with number TVA 15/1890.

## Author Contributions

EL-R, GG-J, LK, MO, and AS-M: conception and design of the work, interpretation of the data, revision of the manuscript, and approval of the submitted version of the manuscript. EL-R, GG-J, and AS-M: drafting of the manuscript. EL-R and GG-J: acquisition and analysis of the data. All authors contributed to the article and approved the submitted version.

## Conflict of Interest

The authors declare that the research was conducted in the absence of any commercial or financial relationships that could be construed as a potential conflict of interest.

## References

[1] Raghu G Collard HR Egan JJ Martinez FJ Behr J Brown KK. ATS/ERS/JRS/ALAT committee on idiopathic pulmonary fibrosis. An official ATS/ERS/JRS/ALAT statement: idiopathic pulmonary fibrosis: evidence-based guidelines for diagnosis and management. Am J Respir Crit Care Med. (2011) 183:788–824. 10.1164/rccm.2009-040GL 21471066 PMC5450933

[2] Noble PW Albera C Bradford WZ Costabel U Glassberg MK Kardatzke D. Pirfenidone in patients with idiopathic pulmonary fibrosis (CAPACITY): two randomised trials. Lancet. (2011) 377:1760–9. 10.1016/S0140-6736(11)60405-4 21571362

[3] Richeldi L Costabel U Selman M Kim DS Hansell DM Nicholson AG. Efficacy of a tyrosine kinase inhibitor in idiopathic pulmonary fibrosis. N Engl J Med. (2011) 365:1079–87. 10.1056/NEJMoa1103690 21992121

[4] Bishop AE. Pulmonary epithelial stem cells. Cell Prolif. (2004) 37:89–96. 10.1111/j.1365-2184.2004.00302.x 14871239 PMC6495778

[5] Myers JL Katzenstein AL. Epithelial necrosis and alveolar collapse in the pathogenesis of usual interstitial pneumonia. Chest. (1988) 94:1309–11. 10.1378/chest.94.6.1309 3191777

[6] Uhal BD Joshi I Hughes WF Ramos C Pardo A Selman M. Alveolar epithelial cell death adjacent to underlying myofibroblasts in advanced fibrotic human lung. Am J Physiol. (1998) 275:L1192–9. 10.1152/ajplung.1998.275.6.L1192 9843857

[7] Selman M Pardo A. Role of epithelial cells in idiopathic pulmonary fibrosis: from innocent targets to serial killers. Proc Am Thorac Soc. (2006) 3:364–72. 10.1513/pats.200601-003TK 16738202

[8] Günther A Schmidt R Nix F Yabut-Perez M Guth C Rosseau S. Surfactant abnormalities in idiopathic pulmonary fibrosis, hypersensitivity pneumonitis and sarcoidosis. Eur Respir J. (1999) 14:565–73. 10.1034/j.1399-3003.1999.14c14.x 10543276

[9] Ochs M. The closer we look the more we see? Quantitative microscopic analysis of the pulmonary surfactant system. Cell Physiol Biochem. (2010) 25:27–40. 10.1159/000272061 20054142

[10] Perez-Gil J Weaver TE. Pulmonary surfactant pathophysiology: current models and open questions. Physiology. (2010) 25:132–41. 10.1152/physiol.00006.2010 20551227

[11] Bachofen H Gehr P Weibel ER. Alterations of mechanical properties and morphology in excised rabbit lungs rinsed with a detergent. J Appl Physiol. (1979) 47:1002–10. 10.1152/jappl.1979.47.5.1002 511700

[12] Sera T Yokota H Tanaka G Uesugi K Yagi N Schroter RC. Murine pulmonary acinar mechanics during quasi-static inflation using synchrotron refraction-enhanced computed tomography. J Appl Physiol. (2013) 115:219–28. 10.1152/japplphysiol.01105.2012 23661619

[13] Mead J Takishima T Leith D. Stress distribution in lungs: a model of pulmonary elasticity. J Appl Physiol. (1970) 28:596–608. 10.1152/jappl.1970.28.5.596 5442255

[14] Froese AR. Shimbori C Bellaye PR Inman M Obex S Fatima S. Stretch-induced activation of transforming growth factor-β1 in pulmonary fibrosis. Am J Resp Crit Care Med. (2016) 194:84–96. 10.1164/rccm.201508-1638OC 26771871

[15] Cabrera-Benítez NE Parotto M Post M Han B Spieth PM Cheng WE. Mechanical stress induces lung fibrosis by epithelial-mesenchymal transition. Crit Care Med. (2012) 40:510–7. 10.1097/CCM.0b013e31822f09d7 21926573 PMC5061566

[16] Sisson TH Mendez M Choi K Subbotina N Courey A Cunningham A. Targeted injury of type II alveolar epithelial cells induces pulmonary fibrosis. Am J Respir Crit Care Med. (2010) 181:254–63. 10.1164/rccm.200810-1615OC 19850947 PMC2817814

[17] Burkhardt A. Alveolitis and collapse in the pathogenesis of pulmonary fibrosis. Am Rev Respir Dis. (1989) 140:513–24. 10.1164/ajrccm/140.2.513 2669580

[18] Serrano-Mollar A Nacher M Gay-Jordi G Closa D Xaubet A Bulbena O. Intratracheal transplantation of alveolar type II cells reverses bleomycin-induced lung fibrosis. Am J Respir Crit Care Med. (2007) 176:1261–8. 10.1164/rccm.200610-1491OC 17641155

[19] Guillamat-Prats R Gay-Jordi G Xaubet A Peinado VI Serrano-Mollar A. Alveolar type II cell transplantation restores pulmonary surfactant protein levels in lung fibrosis. J Heart Lung Transplant. (2014) 33:758–65. 10.1016/j.healun.2014.03.008 25023067

[20] Banerjee ER Laflamme MA Papayannopoulou T Kahn M Murry CE Henderson WR Jr. Human embryonic stem cells differentiated to lung lineage-specific cells ameliorate pulmonary fibrosis in a xenograft transplant mouse model. PLoS ONE. (2012) 7:e33165. 10.1371/journal.pone.0033165 22470441 PMC3314647

[21] How CK Chien Y Yang KY Shih HC Juan CC Yang YP. Induced pluripotent stem cells mediate the release of interferon gamma-induced protein 10 and alleviate bleomycin induced lung inflammation and fibrosis. Shock. (2013) 39:261–70. 10.1097/SHK.0b013e318285f2e2 23364435

[22] Huang K Kang X Wang X. Conversion of bone marrow mesenchymal stem cells into type II alveolar epithelial cells reduces pulmonary fibrosis by decreasing oxidative stress in rats. Mol Med Rep. (2015) 11:1685. 10.3892/mmr.2014.2981 25411925 PMC4270324

[23] Alvarez-Palomo B Sanchez-Lopez LI Moodley Y Edel MJ Serrano-Mollar A. Induced pluripotent stem cell-derived lung alveolar epithelial type II cells reduce damage in bleomycin-induced lung fibrosis. Stem Cell Res Ther. (2020) 11:213. 10.1186/s13287-020-01726-3 32493487 PMC7268474

[24] Serrano-Mollar A. Cell therapy in idiopathic pulmonary fibrosis†. Med Sci. (2018) 6:64. 10.3390/medsci6030064 30104544 PMC6164035

[25] Serrano-Mollar A Gay-Jordi G Guillamat-Prats R Closa D Hernandez-Gonzalez F Marin P. Safety and tolerability of alveolar type II cell transplantation in idiopathic pulmonary fibrosis. Chest. (2016) 150:533–43. 10.1016/j.chest.2016.03.021 27020420

[26] Richards RJ Davies N Atkins J Oreffo VI. Isolation, biochemical characterisation, and culture of lung type II cells of the rat. Lung. (1987) 165:143–58. 10.1007/BF02714430 3108591

[27] Knudsen L Lopez-Rodriguez E Berndt L Steffen L Ruppert C Bates JHT. Alveolar micromechanics in bleomycin-induced lung injury. Am J Resp Cell Mol Biol. (2018) 59:757–69. 10.1165/rcmb.2018-0044OC 30095988 PMC6293074

[28] Hsia CW Hyde DM Ochs M Weibel ER. An official research policy statement of the american thoracic society/european respiratory society: standards for quantitative assessment of lung structure. Am J Resp Crit Care Med. (2010) 181:394–418. 10.1164/rccm.200809-1522ST 20130146 PMC5455840

[29] Ochs M Mühlfeld C. Quantitative microscopy of the lung: a problem-based approach. Part 1: basic principles of lung stereology. Am J Physiol-Lung Cell Mol Physiol. (2013) 305:L15–22. 10.1152/ajplung.00429.2012 23624789

[30] Lutz D Gazdhar A Lopez-Rodriguez E Ruppert C Mahavadi P Günther A. Alveolar derecruitment and collapse induration as crucial mechanisms in lung injury and fibrosis. Am J Resp Cell Mol Biol. (2015) 52:232–43. 10.1165/rcmb.2014-0078OC 25033427

[31] Ochs M Nenadic I Fehrenbach A Albes JM Wahlers T Richter J. Ultrastructural alterations in intraalveolar surfactant subtypes after experimental ischemia and reperfusion. Am J Resp Crit Care Med. (1999) 160:718–24. 10.1164/ajrccm.160.2.9809060 10430751

[32] Parimon T Yao C Stripp BR Noble PW Chen P. Alveolar epithelial type II cells as drivers of lung fibrosis in idiopathic pulmonary fibrosis. Int J Mol Sci. (2020) 21:2269. 10.3390/ijms21072269 32218238 PMC7177323

[33] Mahavadi P Henneke I Ruppert C Knudsen L Venkatesan S Liebisch G. Altered surfactant homeostasis and alveolar epithelial cell stress in amiodarone-induced lung fibrosis. Tox Sci. (2014) 142:285–97. 10.1093/toxsci/kfu177 25163675

[34] Korfei M Ruppert C Mahavadi P Henneke I Markart P Koch M. Epithelial endoplasmic reticulum stress and apoptosis in sporadic idiopathic pulmonary fibrosis. Am J Resp Crit Care Med. (2008) 178:838–46. 10.1164/rccm.200802-313OC 18635891 PMC2566794

[35] Lopez-Rodriguez E Gay-Jordi G Mucci A Lachmann N Serrano-Mollar A. Lung surfactant metabolism: early in life, early in disease and target in cell therapy. Cell Tissue Res. (2017) 367:721–35. 10.1007/s00441-016-2520-9 27783217

[36] Moeller A Ask K Warburton D Gauldie J Kolb M. The bleomycin animal model: a useful tool to investigate treatment options for idiopathic pulmonary fibrosis? Int J Biochem Cell B. (2008) 40:362–82. 10.1016/j.biocel.2007.08.011 17936056 PMC2323681

[37] Kobayashi T Nitta K Ganzuka M Inui S Grossmann G Robertson B. Inactivation of exogenous surfactant by pulmonary edema fluid. Pediatr Res. (1991) 29:353–6. 10.1203/00006450-199104000-00005 1852528

[38] Nitta K Kobayashi T. Impairment of surfactant activity and ventilation by proteins in lung edema fluid. Respir Physiol. (1994) 95:43–51. 10.1016/0034-5687(94)90046-9 8153452

[39] Zasadzinski A Alig TF Alonso C Bernardino de la Serna J Perez-Gil J Taeusch HW. Inhibition of pulmonary surfactant adsorption by serum and the mechanisms of reversal by hydrophilic polymers: theory. Biophys J. (2005) 89:1621–9. 10.1529/biophysj.105.062646 16006630 PMC1366666

[40] Gunasekara L Schoel WM Schürch S Amrein MW. A comparative study of mechanisms of surfactant inhibition. Biochim Biophys Acta- Biomembranes. (2008) 1778:433–44. 10.1016/j.bbamem.2007.10.027 18036553

[41] Lopez-Rodriguez E Ospina OL Echaide M Taeusch HW Pérez-Gil J. Exposure to polymers reverses inhibition of pulmonary surfactant by serum, meconium, or cholesterol in the captive bubble surfactometer. Biophys J. (2012) 103:1451–9. 10.1016/j.bpj.2012.08.024 23062337 PMC3471484

[42] Lopez-Rodriguez E Boden C Echaide M Perez-Gil J Kolb M Gauldie J. Surfactant dysfunction during overexpression of TGF-β1 precedes profibrotic lung remodeling *in vivo*. Am J Physio-Lung Cell Mol Physiol. (2016) 310:L1260–71. 10.1152/ajplung.00065.2016 27106287

