# Supplementary material for: Improved Alveolar Dynamics and Structure After Alveolar Epithelial Type II Cell Transplantation in Bleomycin Induced Lung Fibrosis
Source: Front Med (Lausanne). 2021 Feb 17;8:640020. doi: 10.3389/fmed.2021.640020 (PMC7925848; doi:10.3389/fmed.2021.640020)
Supplement: Supplementary file 1 [file Table_1.DOCX]

***Supplementary Material***

Improved alveolar dynamics and structure after alveolar epithelial type II cell transplantation in bleomycin induced lung fibrosis

Elena Lopez-Rodriguez^1,2*^, Gemma Gay-Jordi^3,4*^, Lars Knudsen^1,5^, Matthias Ochs^1,2,6#^ and Anna Serrano-Mollar^3,4#^

^1^Institute of Functional Anatomy, Charité - Universitaetsmedizin Berlin, Berlin, Germany

^2^Institute of Functional and Applied Anatomy, Hannover Medical School, Hannover, Germany

^3^Experimental Pathology Department, Institut d'Investigacions Biomèdiques de Barcelona, Consejo Superior de Investigaciones Cientificas (IIBB-CSIC) Barcelona, Institut d’Investigacions Biomédiques August Pi i Sunyer (IDIBAPS), Barcelona, Spain

^4^Centro de Investigaciones Biomédicas en Red de Enfermedades Respiratorias (CIBERES), Melchor Fernández Almagro 3, 28029 Madrid, Spain

^5^Biomedical Research in Endstage and Obstructive Lung Disease Hannover (BREATH), Member of the German Center for Lung Research (DZL), Hannover, Germany

^6^German Center for Lung Research (DZL), Berlin, Germany

*both authors contributed equally to the work

**# Correspondence:**Corresponding Authors: Anna Serrano-Mollar, PhD ([anna.serranomollar@iibb.csic.es](mailto:anna.serranomollar@iibb.csic.es)), Matthias Ochs, Prof. Dr. ([matthias.ochs@charite.de](mailto:matthias.ochs@charite.de))

**Keywords: lung fibrosis, alveolar epithelial type 2 cells, lung surfactant, cell therapy, bleomycin, alveolar dynamics, lung structure.**

## Supplementary Figures


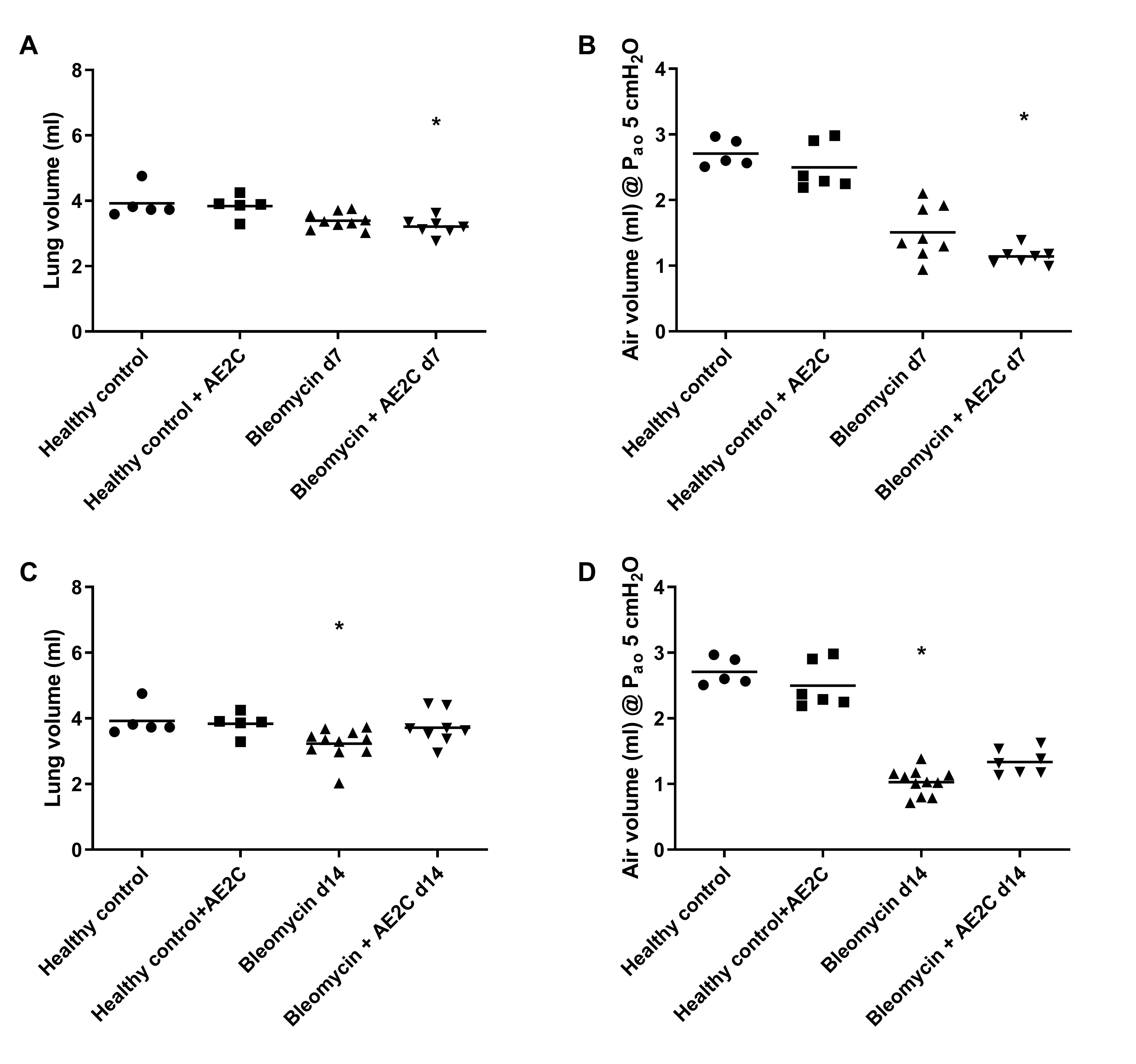


**Supplementary Figure 1.** **Lung and air volume of bleomycin induced lung injury (d7) and fibrosis (d14) after AE2C transplantation**. Top panel (A-B): lung and air volume of bleomycin induced lung injury (7 days after bleomycin application) and AE2C transplantation (3 days after bleomycin application). A) Lung volume measured by fluid displacement at day 7 after bleomycin application. B) Volume of air used to inflate the lungs at a constant airway opening pressure of 5cmH_2_O at day 7 after bleomycin application. Bottom panel (C-D): lung and air volume of bleomycin induced lung fibrosis (14 days after bleomycin application) and AE2C transplantation (7 days after bleomycin application). C) Lung volume measured by fluid displacement at day 14 after bleomycin application. D) Volume of air used to inflate the lungs at a constant airway opening pressure of 5cmH_2_O at day 14 after bleomycin application.


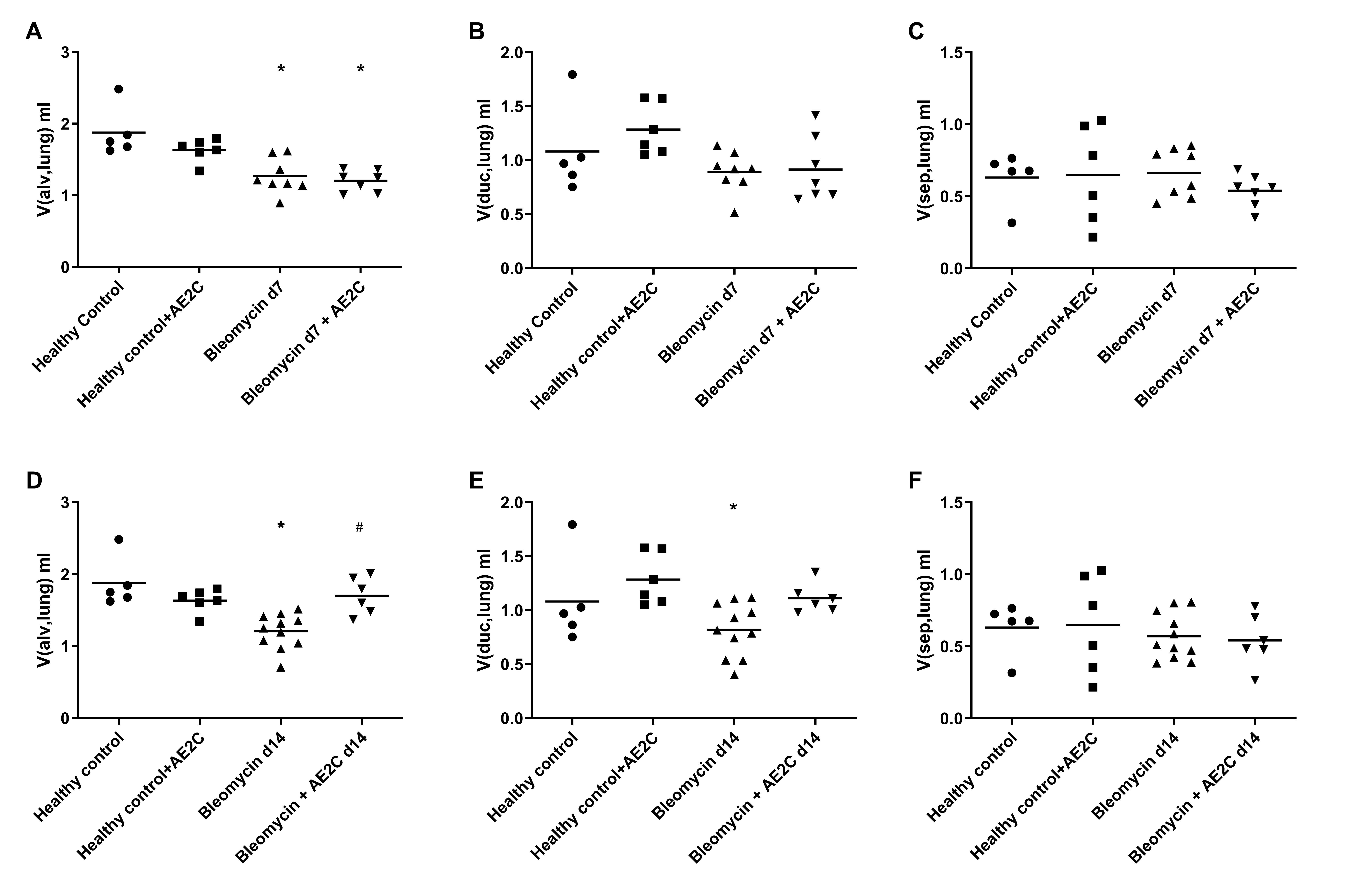


**Supplementary Figure 2.** **Lung parenchyma components volume of bleomycin induced lung injury (d7) and fibrosis (d14) after AE2C transplantation**. Top panel (A-C): lung parenchyma components volume of bleomycin induced lung injury (7 days after bleomycin application) and AE2C transplantation (3 days after bleomycin application). A) Total volume of alveolar spaces per lung at day 7 after bleomycin application. B) Total volume of ductal spaces per lung at day 7 after bleomycin application. C) Total volume of septal tissue per lung at day 7 after bleomycin application. Bottom panel (C-E): lung parenchyma components volume of bleomycin induced lung injury (14 days after bleomycin application) and AE2C transplantation (7 days after bleomycin application). D) Total volume of alveolar spaces per lung at day 7 after bleomycin application. E) Total volume of ductal spaces per lung at day 7 after bleomycin application. F) Total volume of septal tissue per lung at day 7 after bleomycin application.
